# Eliciting the impacts of cellular noise on metabolic trade-offs by quantitative mass imaging

**DOI:** 10.1038/s41467-019-08717-w

**Published:** 2019-02-19

**Authors:** A. E. Vasdekis, H. Alanazi, A. M. Silverman, C. J. Williams, A. J. Canul, J. B. Cliff, A. C. Dohnalkova, G. Stephanopoulos

**Affiliations:** 1 Department of Physics, University of Idaho, Moscow, ID 83844 USA; 2 Department of Chemical Engineering, Massachusetts Institute of Technology, Cambridge, MA 02139 USA; 3 Department of Statistical Science, University of Idaho, Moscow, ID 83844 USA; 4 Environmental Molecular Sciences Laboratory, Pacific Northwest National Laboratory, Richland, WA 99354 USA

## Abstract

Optimal metabolic trade-offs between growth and productivity are key constraints in strain optimization by metabolic engineering; however, how cellular noise impacts these trade-offs and drives the emergence of subpopulations with distinct resource allocation strategies, remains largely unknown. Here, we introduce a single-cell strategy for quantifying the trade-offs between triacylglycerol production and growth in the oleaginous microorganism *Yarrowia lipolytica*. The strategy relies on high-throughput quantitative-phase imaging and, enabled by nanoscale secondary ion mass spectrometry analyses and dedicated image processing, allows us to image how resources are partitioned between growth and productivity. Enhanced precision over population-averaging biotechnologies and conventional microscopy demonstrates how cellular noise impacts growth and productivity differently. As such, subpopulations with distinct metabolic trade-offs emerge, with notable impacts on strain performance and robustness. By quantifying the self-degradation of cytosolic macromolecules under nutrient-limiting conditions, we discover the cell-to-cell heterogeneity in protein and fatty-acid recycling, unmasking a potential bet-hedging strategy under starvation.

## Abstract (web summary)

How cellular noise impacts metabolic trade-offs remains unknown. Here, the authors use a quantitative single-cell mass imaging strategy to reveal that cellular noise impacts cellular biomass and triacylglycerol accumulation, as well as protein and fatty-acid recycling under starvation, differently.

## Introduction

The rewiring of metabolic networks by synthetic biology or adaptive evolution can induce trade-offs between pathways maintaining balanced growth and the production of specific metabolites^1^. Similar to a Pareto front^2^, these trade-offs can be non-optimal with potential implications for the maintenance of genetic diversity^3^ and co-optimization of production titers and yields by metabolic engineering^4^. While these trade-offs are challenging to predict given the underlying interactions between distal and often seemingly unrelated genes^5^, biotechnologies, such as mass spectrometry, can quantify how resources are allocated to growth and the production of specific metabolites^6^. These screening approaches, however, operate in a population-averaging mode, and thus cannot detect the presence of metabolic subpopulations that can emerge due to cellular noise and cytosolic stochastic phenomena^7–11^. These subpopulations can have a significant impact on clonal populations, such as the emergence of phenotypically different subpopulations offering bet-hedging survival strategies under stress^12–14^. Specific to metabolic engineering, overproducing subpopulations with distinct industrial interest can emerge^15,16^, while underproducing subpopulations can be indicative of reduced robustness under changing environmental conditions^9,17^.

Detecting metabolic subpopulations requires single-cell resolution, which, for live cells, can be attained by conventional optical microscopy. However, conventional microscopy typically informs about the cell and product volumes, which, in an energy-balance context, only partially contributes to the cell’s enthalpy^18^. Further, in a mass-balance context, conventional optical methods assume that biomass and product densities are homogeneous between clonal cells and independent of the growth conditions, which, as demonstrated here, is not generally valid. More recently, suspended microfluidic resonators^19^ and cantilever picobalances^20^ have emerged as a means to determine the mass of single-cells. Despite their enhanced sensitivity and temporal resolution, these approaches determine the average mass of individual cells and, as such, are unable to detect and quantify specific intracellular metabolites. Therefore, despite the considerable recent progress in single-cell methods^21–23^, metabolic trade-off phenotyping with single-cell resolution remains an important, yet unmet, biotechnology target.

**Fig. 1:**
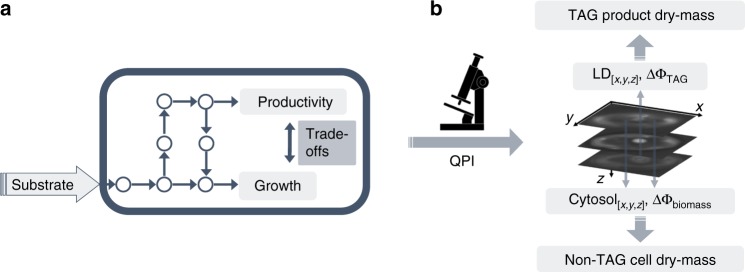
Metabolic trade-offs by quantitative mass imaging. **a** Schematic illustrating substrate uptake and resource partitioning to growth and production, as well as the underlying trade-offs between these two metabolic objectives. **b** Quantitative phase-imaging (QPI) enables the independent localization (*x*,*y*,*z*) and phase-delay quantification of the cell cytosol (ΔΦ_cytosol_) and TAG loaded lipid droplets (ΔΦ_TAG_). ΔΦ_cytosol_ and ΔΦ_TAG_ are subsequently converted to their corresponding dry-mass values, enabling trade-off phenotyping between growth and TAG production

To meet this target, we adapt quantitative phase imaging^24–29^ to the phenotyping of how resources are partitioned between growth and productivity, and thus the resulting trade-offs between these two metabolic objectives with single-cell resolution (Fig. 1a). For this, we use triacylglycerol (TAG) production in *Yarrowia lipolytica* as a model process^6^ (Fig. 1b). We select *Y. lipolytica*, an obligate aerobic, oleaginous yeast for its importance in the production of biofuel precursors. To this end, *Y. lipolytica* has recently attracted substantial attention due to its compatibility with genetic engineering and innate capability to accumulate substantial amounts of TAGs^6,30,31^.

By capturing the optical-phase delay (ΔΦ) of the cell cytosol and TAG loaded lipid droplets (LDs), we obtain the corresponding dry-mass values, and thus the metabolic trade-offs between growth (cytosol) and productivity (TAGs). We confirm the validity of this approach with nanoscale secondary ion mass spectrometry (NanoSIMS)^32^ and report that quantitative mass imaging exhibits more than 55% higher precision than conventional microscopy in growth and productivity phenotyping. With dedicated image processing, we perform growth-productivity bivariate analyses^33^ and discover that the growth and productivity robustness to cellular noise are not interrelated under various conditions. As such, subpopulations exhibiting different metabolic trade-offs emerge with significant impacts on the overall performance of clonal populations. These impacts include a previously unobserved cell-to-cell heterogeneity in macromolecule recycling under starvation, possibly indicating the presence of a bet-hedging strategy under nutrient-limiting conditions.

## Results

### Imaging strategy

**Fig. 2:**
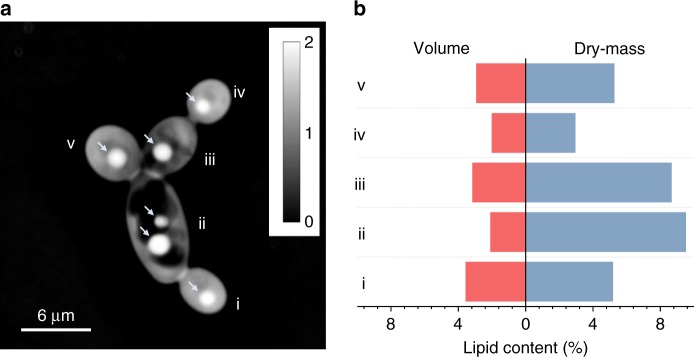
Quantitative mass imaging and cell-to-cell lipid-content heterogeneity. **a** An optical-phase image of individual *Y. lipolytica* cells labeled from (i) to (iv); arrows indicate the cytosolic LDs, and scale-bar is displayed in radians. **b** Histogram of the lipid-content in % volume (V_TAG_/V_biomass_) and dry-mass (DM_TAG_/DM_biomass_) ratios for the cells shown in **a**; importantly, the single-cell volumetric lipid-content is seen to scale inversely with the DM lipid-content specifically for cells (i), (ii), and (iii)

To perform quantitative mass imaging, we introduced 2 µL of a growing *Y. lipolytica* culture between two coverslips without further processing. The sample was subsequently transferred to an automated microscope equipped with quantitative phase and fluorescent imaging (Methods). For quantitative phase imaging, we employed spatial light interference microscopy (SLIM) by projecting the phase-contrast intensity images onto a spatial light modulator and applying additional phase-delays to the non-diffracted wavefront (background) with respect to the diffracted wavefront (cell)^24^. In this way, we were able to capture both the size and optical-phase delay of the cell cytosol (ΔΦ_cytosol_) and TAG product (ΔΦ_TAG_) at the single-cell level (Figs. 1b and 2a).

**Fig. 3:**
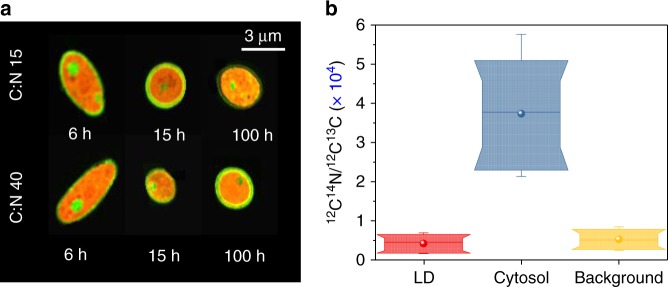
Elemental composition of *Y. lipolytica*. **a** NanoSIMS images of the MTYL038 strain at two C/N growth conditions for 6, 15, and 100 h (Methods); the cytosolic pools of naturally abundant ^14^N and the LD content of ^13^C are highlighted in red and green, respectively. **b** Box-plots of ^12^C^14^N/^12^C^13^C ratio of the cytosolic LDs droplets (red), the cytosol excluding the LDs (blue), and the extracellular background (yellow) for 40 individual single-cell and single-LD observations, for cells sampled at C/N:15 and C/N:40 at 6, 15, and 100 h (Methods). Specifically, n_C/N: 15, 6 h_ = 8, n_C/N: 15, 15 h_ = 2, n_C/N: 15, 100 h_ = 2, n_C/N: 40, 6 h_ = 12, n_C/N: 40, 15 h_ = 4, n_C/N: 40, 100 h_ = 12. Box-plots represent the 10th, 25th, 75th, and 90th percentile, whiskers represent the 5th and 95th percentile, while the median and mean values of the ensemble distribution are indicated by the horizontal line and sphere, respectively

Subsequently, we converted ΔΦ_cytosol_ and ΔΦ_TAG_ to their corresponding dry-mass (DM) values, thus attaining the cell-to-cell lipid-content heterogeneity in both volume and DM ratios (Fig. 2). To complete this conversion, we hypothesized that the cell cytosol is primarily comprised of proteins and nucleic acids, dispersed with LDs that are loaded with TAGs at a negligible protein content. We confirmed this hypothesis by characterizing the cytosolic and LD elemental composition with NanoSIMS^32^. Indeed, after exposure to U-^13^C glucose at various carbon-to-nitrogen ratios (C/N) and durations (Methods) using two independent cultures, we found the cytosol to be uniformly comprised of naturally abundant nitrogen (^14^N), as illustrated in Fig. 3a. Similarly, LDs, which were co-localized by Transmission Electron Microscopy (TEM) via osmium staining and NanoSIMS (Supplementary Fig. 1), were found to be comprised primarily of ^13^C and a comparable ^14^N content to the extracellular background (Fig. 3b).

### Image analysis

To convert ΔΦ_cytosol_ to the corresponding DM, we employed the protein-specific refractive index increment^24–28^ (Methods). However, unlike the cell cytosol that can be approximated as an aqueous protein solution, glycerolipids self-assemble into spherical LDs^34^, which for *Y. lipolytica* are relatively homogeneous with approximately 90–95% TAGs^35^. As such, it is challenging to apply the specific refractive index increment to LDs. To address this, we employed the experimentally determined LD refractive index and applied the Clausius-Mossotti equation to determine the number-density of TAG molecules (N_TAG_)^36^. N_TAG_ was subsequently converted to the corresponding LD mass density (Methods). For *Y. lipolytica*, we considered a mixture of triolein, stearin, tripalmitin, trilinolein, and tripalmitolein^35^, yielding a polarizability parameter of 1.048 × 10^−22^ cm^−3^ and 1.437 × 10^−21^ gr molecular weight (Methods). Generally, the polarizability and molecular weight parameters are not expected to vary significantly under typical growth conditions. Indicatively for *Y. lipolytica*, the mixture polarizability and molecular weight change by approximately 0.45% and 0.28% under N-limited and C-limited conditions using glucose as the sole carbon source^31^. For a wider range of substrates and growth durations, we found that the mixture polarizability and molecular weight parameters change on average by 0.6% (1.8% maximum change) and 0.6% (1.6% maximum change), respectively, for various *Y. lipolytica* strains (see Methods and Supplementary Tables 1 and 2 for further information).

**Fig. 4:**
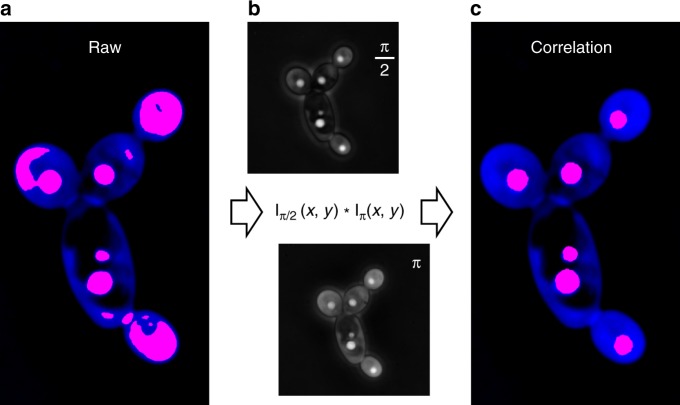
Image processing by spatial cross-correlation. **a** The quantitative-phase image shown in Fig. 2a overlaid with the thresholded areas that exhibit phase-delay values (ΔΦ) comparable to the LDs; the thresholded areas (red) include both parts of the cytosol and the LDs, given their similarity in ΔΦ. **b** The spatial cross-correlation of the π/2 and π phase-modulated intensity images eliminated the cytosolic background contribution, enabling the error-free localization of the LDs by intensity thresholding (**c**)

To determine the ΔΦ_cytosol_ and ΔΦ_TAG_ of individual cells, we processed the acquired images and localized the cell-contour and LDs via their ΔΦ levels without any fluorescent labeling. However, while the cytosol-to-background contrast was adequate for cell-segmentation^37^, the LD-to-cytosol contrast (i.e., ΔΦ_TAG_/ΔΦ_cytosol_) was insufficient for automated thresholding. To overcome this, we also collected phase-contrast intensity images, where we additionally modulated the cytosol and LD diffracted wavefronts^24^ by π/2 and π (Methods). Cross-correlating the resulting images suppressed the cytosolic signal and thus the non-specific contributions to the LD localization (Fig. 4). Overall, this approach yielded greater than 98% agreement with fluorescence-based LD localization^26^ (Supplementary Fig. 2), and accelerated image processing at rates that enabled more than 10^3^ single-cell observations per experimental condition.

### Comparison with conventional microscopy

**Fig. 5:**
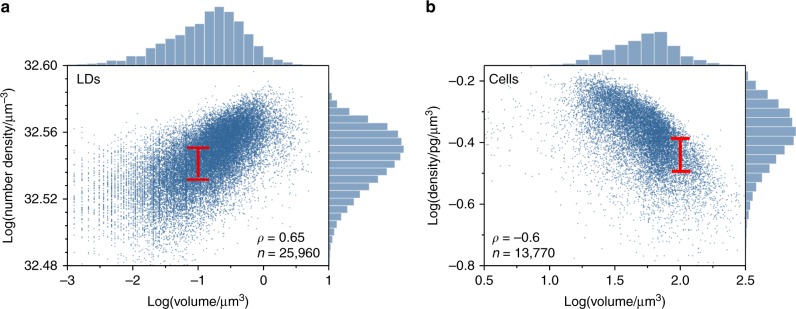
Comparison between quantitative-mass with conventional imaging. **a** Scatter plot and marginal histograms of the TAG number density per lipid droplet (LD) as a function of the LD volume for various Y. lipolytica strains and growth conditions (Methods); red line indicates the interquartile range (IQR) for an LD volume of 0.1 µm^3^, and inset illustrates the number of observations (n), the Spearman correlation coefficient (ρ), yielding *p* < 0 .001. **b** A similar scatter plot for the non-TAG cell dry-density as a function of the cell volume with the red line indicating the IQR for a cell volume of 100 µm^3^; inset illustrates the number of observations (n), the Spearman correlation coefficient (ρ), yielding *p* < 0.001. The data represent the ensemble of 5 different experimental conditions, each performed in triplicates (see Methods), including: MTYL038_17hr_ (n_A_ = 400, n_B_ = 416, n_G_ = 535 single-cell observations), MTYL038_28hr_ (n_A_ = 664, n_B_ = 566, n_G_ = 686), MTYL038_52hr_ (n_A_ = 524, n_B_ = 584, n_G_ = 762), MTYL038_76hr_ (n_A_ = 471, n_B_ = 825, n_G_ = 695), MTYL038_100hr_ (n_A_ = 633, n_B_ = 616, n_G_ = 941), MTYL038_124hr_ (n_A_ = 655, n_B_ = 793, n_G_ = 894), and Po1g_100hr_ (n_A_ = 685, n_B_ = 748, n_G_ = 677), yielding a total of 13,770 single-cell and 25,960 single LD observations

Using phase-imaging, we compared the volume with the TAG number-density of approximately 25,960 individual LDs of single *Y. lipolytica* cells grown under 7 different conditions, including 3 independent replicate cultures per condition (Methods). We found that the LD volume (V_TAG_) correlated positively but moderately with the TAG number-density (N_TAG_) at various growth and strain conditions, with an overall Spearman correlation coefficient of *ρ* = 0.65 (*p* < .001) (Fig. 5a). As such, N_TAG_ and V_TAG_ did not exhibit identical dynamics at all timepoints. For example, during the 17–28 h period of MTYL038 at C/N: 150, both the N_TAG_ and V_TAG_ increased (one-sided *t*-test, *p* = 0.02 for both variables). In contrast, during the 76–124 h period, strong evidence supported that V_TAG_ increased in volume (one-sided *t*-test, *p* = 0.01); however, similarly strong evidence was not detected for N_TAG_ (one-sided *t*-test, *p* = 0.2) (Supplementary Fig. 3). Under the same conditions, we imaged the volume and dry-mass of approximately 13,770 individual *Y. lipolytica* cells. Through this analysis, we found that the cell dry-density correlated negatively but moderately with cell volume (Spearman correlation coefficient *ρ* = −0.6, (*p* < .001, Fig. 5b), and largely independent of growth and strain conditions (Supplementary Fig. 4).

In addition to the weak correlations, we noted significant variability in the TAG product and cell density relationships with their sizes (Fig. 5). Indicatively, a cell volume of 100 µm^3^ corresponded to a median dry-density of 0.37 pg/µm^3^ with a 0.1 pg/µm^3^ interquartile range (IQR) (Fig. 5b). Similarly, an LD volume of 0.1 µm^3^ corresponded to a median TAG number density of 3.4 × 10^32^ µm^−3^ with an IQR of 1.4 × 10^31^ µm^−3^ (Fig. 5a). These levels of variability are indicative that the size-density relationship for both the TAG product and non-TAG biomass are subject to cellular noise and thus cell-to-cell heterogeneity in a clonal population. In addition to the significant biophysical insight, this finding unmasks the challenge in attaining a global, deterministic, correction factor to the size-density relationship.

Both the weak correlations and increased variability in the density-size relationship for TAG product and non-TAG biomass unmask potential precision limitations of conventional, volumetric microscopy in phenotyping growth and productivity at the single-cell level. These limitations pertain both to precision phenotyping of the population response and underlying cell-to-cell phenotypic heterogeneity (Fig. 2b). For *Y. lipolytica* specifically, we determined an average error of 55% in TAG content phenotyping, which exhibited statistically significant differences between specific strain and growth-conditions (Supplementary Fig. 5, Supplementary Table 3).

### Growth-productivity trade-offs

To probe the growth-productivity trade-offs with single-cell resolution, we performed a bivariate analysis of growth (non-TAG cell biomass) and productivity (LD TAG-content) for two genetically similar strains (MTYL038 and Po1g, Methods). Specifically, we compared the growth and TAG production at a C/N of 150 for MTYL038 at the 17, 100, and 124 h timepoints (i.e., MTYL038_17_, MTYL038_100_, and MTYL038_124_), and of Po1g at 100 h (Po1g_100_). The growth curves for both strains are shown in Supplementary Figs. 6 and 7.

**Fig. 6:**
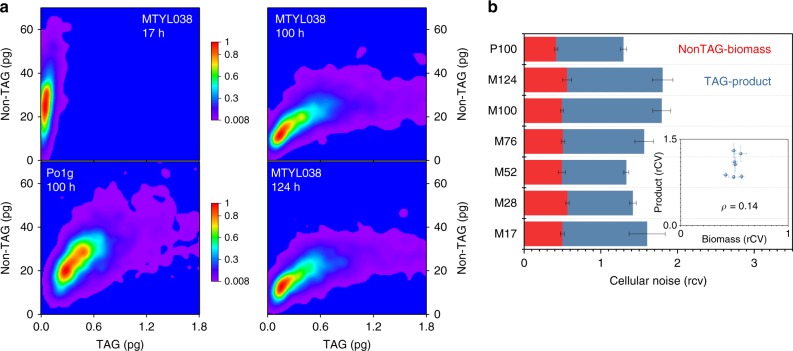
Single-cell growth-productivity trade-offs. **a** 2D probability distributions illustrating the resource allocation strategies between non-TAG biomass and TAG product for MTYL038 grown at C/N:150 for 17, 100, and 124 h, and Po1g at C/N:150 for 100 h. Each distribution represents the ensemble of three biological replicates, and is portrayed with the color scales noted in the figure. **b** The cellular noise in non-TAG biomass (red) and TAG product (blue) quantified via the cell-to-cell phenotypic heterogeneity and the robust coefficient of variation (rCV) for MTYL038_17hr_ (M17), MTYL038_28hr_ (M28), MTYL038_52hr_ (M52), MTYL038_76hr_ (M76), MTYL038_100hr_ (M100), MTYL038_124hr_ (M124), and Po1g_100hr_ (P100). Bars and error-bars indicate the mean and standard-error between three biological replicates, respectively. Under all tested conditions, TAG noise exhibited higher values than growth noise (single-sided t-test between each replicate’s rCV, *p* < 0.025 for all reported conditions). Further, strong evidence supported that production noise is affected by time following the onset of TAG production (28 h) for MTYL038 (one-way ANOVA, F(4,10) = 4.73 and *p* = 0.02); no such evidence was detected for growth noise (one-way ANOVA, F(4,10) = 0.92 and *p* = 0.5). TAG and non-TAG rCV for M100 and P100 were also found to be different (paired-sample *t*-test, *p* = 0.05 for both TAG and non-TAG). Inset plots the dependence of the product rCV on the non-TAG biomass rCV, yielding a Spearman correlation coefficient of 0.14 (*p* = 0.76). Source data of Fig. 6a are provided as a Source Data file

This analysis, illustrated in the 2D probability distributions of Fig. 6a, revealed the distinct resource allocation strategies per medium and strain conditions. The early timepoint of 17 h (mid-exponential growth phase, Supplementary Fig. 6) programmed MTYL038 to allocate resources primarily to growth, as manifested by the increased probability of high non-TAG biomass and low TAG macrostates^35^. In contrast, the later 100 h and 124 h timepoints (stationary phase, Supplementary Fig. 6) programmed MTYL038 to alter its growth-productivity trade-off strategy, manifested by the increased presence of high TAG and low non-TAG biomass macrostates (Fig. 6a). This shift in metabolic trade-offs is attributed to the onset of stationary phase and growth cessation, which typically coincides with the depletion of N levels. Further, a distinct difference between the two strains (MTYL038_100_ and Po1g_100_) was detected. TAG production in MTYL038_100_ was manifested through sporadic high-product and low-biomass (non-TAG) subpopulations (Fig. 6a), which is indicative of increased cellular noise, as further detailed in the following paragraph. In contrast, Po1g_100_ displayed more balanced TAG production. This is evidenced by the co-increased levels of high TAG and non-TAG biomass macrostates (shown in red in Fig. 6a) and the more evenly distributed subpopulations along both the TAG product and non-TAG biomass axes (shown in purple in Fig. 6a).

To further quantify the impact of cellular noise on the growth-production trade-offs, we determined the cell-to-cell heterogeneity in non-TAG biomass and TAG content via the robust coefficient of variation (rCV). Overall, TAG production noise exhibited higher levels than growth noise for all tested experimental conditions (Fig. 6b). This is not surprising given that metabolic networks are thought to have evolved optimal flux distributions for balanced growth^1,38^. Further, growth noise was found to be generally decoupled from production noise, as shown in the inset of Fig. 6b. For example, following the onset of TAG production (28 h, see later sections), TAG production noise in MTYL038 displayed a statistically significant dependence on time; however, no such evidence was found for growth noise during the same period (Fig. 6b). Interestingly, Po1g_100_ exhibited a lower TAG production noise than MTYL038_100_ (rCV_P100-TAG_ = 0.87 ± 0.04 and rCV_M100-TAG _= 1.30 ± 0.12, mean ± standard error between 3 biological replicates). Given the inverse relationship between robustness and noise (R = 1−rCV)^39^, this indicates that this strain exhibits higher production robustness against intracellular and extracellular perturbations (Fig. 6b). This was likely triggered by the medium supplementation with leucine (Methods), a lipogenesis effector amino acid that activates putative leucine degrading and acetyl-CoA-generating pathways^40–42^.

**Fig. 7:**
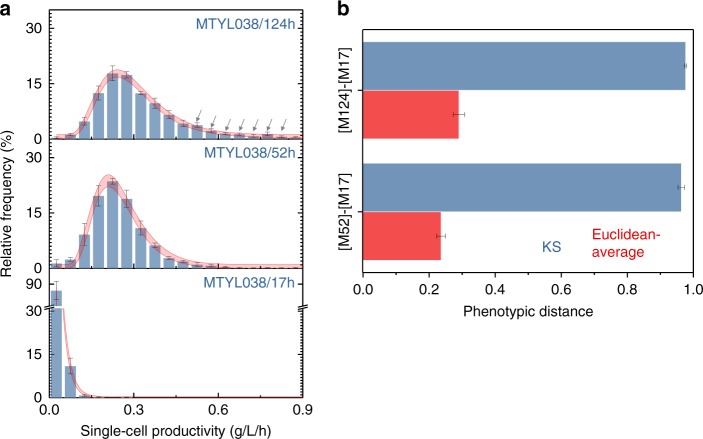
Strain classification by quantitative mass imaging. **a** Single-cell productivity distributions and concatenated non-linear fits for MTYL038_17_ (fit: dF = 56, adj-R^2^ = 0.99, red-x^2^ = 1.71), MTYL038_52_ (fit: dF = 56, adj-R^2^ = 0.94, red-x^2^ = 3.53), and MTYL038_124_ (fit: dF = 56, adj-R^2^ = 0.95, red-x^2^ = 1.75). Bars and error-bars indicate the mean and standard-error between three biological replicates respectively, while gray arrows indicate the presence of overproducing MTYL038_124_ subpopulations. In red, the 95% confidence band for each concatenated fit is shown. Strong evidence supported the temporal dependence of productivity at these timepoints (one-way ANOVA, F(2,6) = 116 and *p* < 0.001). **b** The Kolmogorov-Smirnov (KS) and Euclidian-average distances of the [MTYL038_17_–MTYL038_52_] and [MTYL038_17_–MTYL038_124_] pairs. Bars and error-bars indicate the mean and standard-error between three biological replicates, respectively. Strong evidence supported that the Euclidean-average distances between pairs are different (paired-sample *t*-test, *p* = 0.02); no such evidence was observed for the KS distances (paired-sample *t*-test, *p* = 0.27). Source data are provided as a Source Data file

Capturing the impacts of cellular noise on growth and productivity by population-averaging biotechnologies is challenging^23^. This is indicated, for example, by strong evidence that cell densities (OD, Supplementary Fig. 6) of MTYL038 differ between the 17 h, 28 h, and 52 h timepoints (one-way ANOVA, F(2,6) = 130.3, *p* < 0.001). In contrast, such evidence was not found for growth robustness^9^ (one-way ANOVA F(2,6) = 1.41, *p* = 0.32), as uniquely quantified by mass imaging. Further, cellular noise not only impacts robustness but can also have significant consequences on the overall performance of a clonal population. As such, population-averaging can potentially limit the precision in strain classification, especially for genetically similar strains with reduced phenotypic distance between them. To this end, we probed the productivity (i.e., TAG content per unit volume and time) at the single-cell level using quantitative mass phenotyping. The productivity comparisons of MTYL038_17_ with MTYL038_52_, and MTYL038_124_ are illustrated in Fig. 7a (and Supplementary Fig. 8) as an example. Using the Kolmogorov–Smirnov (KS) statistic to account for cell-to-cell heterogeneity^43–45^, we determined that the phenotypic distances of the [MTYL038_17_–MTYL038_52_] and [MTYL038_17_–MTYL038_124_] pairs do not differ (Fig. 7b). In contrast, the Euclidian-average distances^46^, essentially simulating the readout of conventional, population-averaging methods, were found to be different for the same pairs (Fig. 7b). We attribute this inconsistency to the enhanced precision of single-cell quantitative mass imaging in describing more accurately the origins of overproduction in MTYL038_52_ and MTYL038_124_. Specifically, while the most likely phenotypes of both MTYL038_52_ and MTYL038_124_ exhibited a notable productivity increase in comparison to MTYL038_17_, MTYL038_124_ additionally exhibited a significant overproducing subpopulation (indicated by arrows in Fig. 7a). The presence of these subpopulations was uniquely captured by single-cell quantitative-mass imaging, thus enabling enhanced precision in comparison to population-averaging biotechnologies.

### Trade-offs and heterogeneity under starvation

**Fig. 8:**
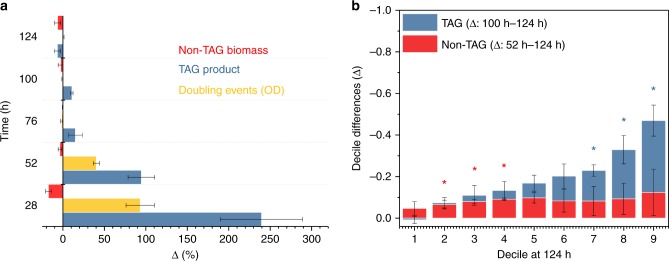
Trade-offs, and heterogeneity under starvation. **a** The time-dependent change of cell non-TAG biomass, TAG product, and cell-doubling (estimated from bulk optical density measurements, Supplementary Fig. 6) for MTYL038 at C/N: 150. Bars and error-bars indicate the mean and standard-error between three biological replicates, respectively. Using repeated measure ANOVA, a statistically significant effect of time was noted for Δ[TAG] (Greenhouse Geiser adjusted p_GG_ = 0.05 with adjustment ε_GG_ = 0.259) for Δ[OD] (p_GG_ = 0.03 with ε_GG_ = 0.253), and to a lesser degree for Δ[non-TAG] (p_GG_ = 0.1 with ε_GG_ = 0.380). **b** Decile differences (Δ) computed using the shift function for the TAG product (blue, 100–124 h period) and non-TAG biomass (red, 52–124 h period) as a function of the corresponding decile at 124 h. Asterisks denote statistically significant decile decreases (one-sided *t*-test, *p* < 0.04), with bars and error-bars indicating the mean and standard-error between three biological replicates, respectively. Source data are provided as a Source Data file

An unexpected metabolic strategy was unmasked for MTYL038 when transferred from rich YPD to a defined medium of C/N: 150 (Methods). During cell-doubling (Supplementary Fig. 6), cell biomass (non-TAG) decreased continuously with the TAG content continuously increasing, especially after the onset of stationary phase (28 h) (Fig. 8a and Supplementary Fig. 9). In contrast, both cell biomass (non-TAG) and the TAG product decreased notably (Fig. 8a and Supplementary Fig. 9) during stationary phase. Specifically, non-TAG biomass decreased (one-sided *t*-test *p* = 0.03) during the 52–124 h period (Supplementary Fig. 6). Similarly, the TAG product decreased (one-sided *t*-test *p* = 0.05) during the 100–124 h period (Supplementary Fig. 6). These findings suggest the activation of an autophagy-based central catabolic process for cell non-TAG biomass^47^ and stored TAGs^48^ following the completion of cell doubling and during late stationary phase, respectively. Importantly, similar conclusions could not be deduced by conventional, volumetric microscopy (Supplementary Fig. 10). This is because, no evidence indicating that cell size changes between the 52 h and 124 h timepoints (paired-sample *t*-test, *p* = 0.2) was found, contrary to the non-TAG cell dry mass at the same timepoints (paired-sample *t*-test, *p* = 0.06). TAG hydrolysis and the breakdown of cytosolic components by autophagy have been previously shown to be interrelated under nutrient deprivation conditions^49^. We anticipate similar nutrient-limiting conditions in our experiments during late stationary phase^35^. With mass imaging, we determined the relative fluxes in single living cells between non-TAG biomass and TAG catabolic pathways, with the two not exhibiting significant differences (paired-sample *t*-test, *p* = 0.02) (Fig. 8a). This finding indicates that *Y. lipolytica* maintenance costs under nutrient starvation induce comparable degradation rates between protein-based cellular components and free fatty acids via TAG hydrolysis.

Further, single-cell resolution unmasked that these catabolic processes do not occur homogeneously within the clonal population, but that they rather exhibit a cell-to-cell heterogeneous response. This is illustrated in Fig. 8b plotting the decile differences for TAG and non-TAG at 100 h and 52 h as a function of the corresponding deciles at 124 h. The decile differences were computed using the shift function and the Harrell-Davis quantile estimator and averaged over the three biological replicates^50,51^. Specifically for TAG, a non-linear shift function was identified (Fig. 8b). This non-linearity indicates that not all deciles (and thus the underlying clonal subpopulations) respond similarly to nutrient limitation (100–124 h). Further, strong evidence supported that only obese subpopulations (7th–9th decile) undergo significant TAG-content decrease (Fig. 8b). In contrast, non-TAG biomass exhibited only a moderate asymmetry of lower magnitude than TAG, with the biomass subpopulations of the 2nd to 4th decile undergoing a statistically significant decrease (Fig. 8b) during stationary phase (52–124 h). These findings indicate that while both TAG and non-TAG autophagy-based catabolism exhibit cell-to-cell heterogeneity, TAG hydrolysis appears to exhibit a stronger non-linear response among subpopulations. Our discovery of cell-to-cell heterogeneity in autophagy-based macromolecule recycling offers unique insight into the existence of sporadic TAG and non-TAG biomass subpopulations, which could potentially act as a bet-hedging survival strategy^14,52^ under nutrient limitation.

## Discussion

Single-cell biology has led to a plethora of unexpected discoveries^7–13,15^, primarily catalyzed by advances in quantifying the growth-rate, genome, transcriptome, and protein content of individual cells^19–23,53–56^. However, the absence of suitable methods for probing how single-cells balance resources between metabolic pathways has masked the impacts of cellular noise on metabolic trade-offs. As such, it remains unknown whether cytosolic or extracellular heterogeneity and underlying stochastic phenomena impose the emergence of subpopulations with distinct resource allocation strategies between growth and the production of specific metabolites.

To address this shortcoming, we developed a single-cell mass phenotyping strategy and applied it to quantify the trade-offs between TAG production and growth in *Y. lipolytica*. The strategy requires only 2 µL of a cell suspension with no further processing, such as washing or staining. With this approach, we attained ~40 cells/min throughput rates for low cell densities and up to ~80 cells/min at increased cell densities. In both cases, cells are imaged in their native medium, thus enabling minimal perturbation to the culture during screening. Using quantitative-phase imaging (Fig. 1), we captured and converted the optical-phase of the cytosol and TAG-loaded LDs of individual cells to their corresponding dry-mass. To complete this conversion, we approximated the cell cytosol as a uniform protein solution and the LDs as organelles uniformly packed with TAGs. We confirmed this approximation by NanoSIMS and TEM correlative imaging under various conditions (Fig. 3). Our NanoSIMS analysis also revealed that *Y. lipolytica* LDs display a relatively negligible protein content, which can exhibit significant diversity for some organisms^57,58^.

Dedicated image acquisition enabled the independent control of the LD and cytosol optical transmission, while correlative image processing aided the localization of these compartments in single-cells (Fig. 4). Using this approach, we performed the high-throughput bivariate screening of non-TAG biomass and TAG production and discovered that cellular noise impacts growth and lipid biogenesis differently. This finding indicates that the robustness of these processes to intracellular perturbations is decoupled in *Y. lipolytica* under the tested conditions (Fig. 6b). As a result, sporadic subpopulations with distinct metabolic trade-offs emerge between isogenic cells at magnitudes and phenotypic positions that depend on growth and strain conditions (Fig. 6a). Further, we employed single-cell mass imaging to quantify autophagy-based catabolic fluxes of non-TAG biomass and TAG content independently in single living cells under nutrient-limiting conditions (Fig. 8a). In comparison to existing approaches relying on gene-encoded fluorescent markers (e.g., the LC3 protein), the developed method can independently quantify protein and fatty-acid catabolic fluxes in a label-free fashion and thus is not subject to biological noise and gene expression fluctuations, and does not require cell permeabilization^59–61^. Importantly, single-cell analyses revealed that autophagy-based catabolic processes occurred primarily for sporadic TAG and non-TAG biomass subpopulations (Fig. 8b). This finding unmasks a possible bet-hedging survival strategy under starvation empowered by cell-to-cell heterogeneity in protein and fatty-acid recycling, similar to the previously identified role of LD heterogeneity against lipotoxicity^62^.

Quantitative mass imaging also revealed two key limitations of existing phenotyping methods. First, it revealed that the size of the TAG product and cell is a poor approximation of their dry-density (Fig. 5), unmasking a precision limitation of conventional microscopy in metabolic trade-off phenotyping (Fig. 2b). This was also pertinent to autophagy-based macromolecule recycling, where, unlike dry-mass, cells did not exhibit significant changes in volume under starvation (Supplementary Fig. 10). Second, single-cell mass phenotyping uniquely unmasked that sporadic subpopulations with distinct metabolic trade-offs play a critical role in the overall population response (Fig. 7a). Therefore, by not detecting these subpopulations, population-averaging biotechnologies are not only unable to inform about strain robustness but may also impede precision strain classification, especially for strains with reduced phenotypic distance between them (Fig. 7b).

In conclusion, we described quantitative single-cell mass imaging for the high-throughput screening of the growth-productivity metabolic trade-off, and its application to the precision strain classification, as well as robustness and autophagy-based catabolic flux quantification. By enabling access to phenotypic information that is otherwise inaccessible by conventional methods, we anticipate that the paradigm of metabolic trade-off imaging will meet several needs in bioengineering and human-health^2–4,63^, beyond TAG biogenesis as probed by way of example here. The combination of mass with fluorescence imaging can enable the screening of alternative metabolites^64^, as well as spark further discoveries in the field of autophagy^59^.

## Methods

### Imaging

Images were acquired with a quantitative-phase imaging system (Phi Optics), based on spatial light interference microscopy (SLIM)^24^. In brief, phase images are formed by projecting the back focal of a phase contrast objective onto a liquid crystal phase modulator, which shifts the optical-phase of the light wavefront scattered by the sample relative to the un-scattered light. Using this approach, we acquired images informing about the relative phase delay of the cells and their cytosolic components (scattered wavefront) with respect to the background (un-scattered wavefront)^24^. The quantitative phase imaging system was coupled to an inverted microscope (DMi8, Leica) equipped with phase contrast and fluorescent imaging capabilities, as well as an automated XYZ stage and a ×100 magnification objective (NA 1.3, PH3, Leica). Images were acquired using the Orca Flash 4.0 camera (Hamamatsu) with a 6.5 µm pixel. The acquisition parameters were set at a 20 ms exposure, and a 20 ms refresh rate for the spatial light modulator that, similar to our previous report, exhibited an ideal combination between stability and temporal resolution^37^. 3D images were acquired by scanning the objective along the imaging path with a step of 400 nm (*Z*-axis), and the stage in the XY plane until ~2000 single-cell observations (approximately 40–60 3D stacks) were acquired on average per experimental condition. Five types of 3D images were collected per stage position. First, phase contrast intensity images, formed by the interference between the portions of the transmitted wavefront through the sample that scatters from the cells and remains un-scattered from the uniform background [I_o_(*x*,*y*,*z*)]. Second, phase contrast intensity images, where an additional phase modulation of ΔΦ_m_ = π/2 was imposed on the scattered wavefront from the sample [I_π/2_(*x*,*y*,*z*)]. Third, phase contrast images, where an additional phase modulation of ΔΦ_m_ = π was imposed on the diffracted wavefront from the sample [I_π_(*x*,*y*,*z*)]. Forth, optical-phase images informing about the phase-delay induced by the cell structure with respect to the background [ΔΦ(*x*,*y*,*z*)]. Fifth, fluorescent images using an incoherent excitation centered at 480 nm [I_f_(*x*,*y*,*z*)]. Upon acquisition, all acquired stack images were transferred to a storage server until further processing.

### Quantitative phase image analysis

Image processing was performed using ImageJ (National Institutes of Health), Matlab (Matlab R2018a, Mathworks), and MetaMorph (MetaMorph Version 7.8.13.0, Molecular Devices). All image processing functions were executed in desktop computers equipped with 32GB RAM. The first step in the image analysis procedure was to detect the cell contour in the optical phase images by direct optical-phase thresholding and no additional pre-processing using the maximum-entropy algorithm in ImageJ^37^. To this end, we selected the *z*-plane of the optical-phase images [ΔΦ(*x,y,z*)] corresponding to the maximum cell area, followed by thresholding using the maximum entropy algorithm (ImageJ). In this way, the cell’s Regions-of-Interest (ROIs) were identified, recorded, and assigned a unique identity per individual cell. Due to the lower axial resolution (*z*-axis) of the imaging set-up than the lateral one (*xy*-plane), we employed a 2D projection method to obtain the volume of all individual cells^9^. To this end, the cell ROIs were fitted with an ellipse and the cell volume was reconstructed as an ellipsoid using the ellipse major and minor axes^65^.

The second step in the image analysis procedure was to localize the cytosolic lipid droplets (LDs). Direct phase-amplitude thresholding gave insufficient discriminatory power due to the significant contributions from the cell wall and cytosol (Figs. 2a and 4). To overcome this, we first 3D deconvoluted the phase-modulated intensity images [I_π/2_(*x,y,z*) and I_π_(*x,y,z*)] using the Point-Spread-Function AutoQuant X_3_ procedure (MetaMorph). The throughput rate of this (automated) step was approximately 12–14 h per experiment. Subsequently, the two deconvoluted images were cross-correlated in Matlab using the following expression:^66^

1 $${\mathrm{C}}\left( {x,y,z} \right) = \frac{{\frac{{{\mathrm{I}}_{\frac{{\mathrm{\pi }}}{2}}(x,y,z)}}{{{\mathrm{I}}_{\frac{{\mathrm{\pi }}}{2} {\vdots} {\mathrm{max}}}}} \cdot \frac{{{\mathrm{I}}_{\mathrm{\pi }}(x,y,z)}}{{{\mathrm{I}}_{{\mathrm{\pi }}\vdots{\mathrm{max}}}}}}}{{[\frac{{{\mathrm{I}}_{\frac{{\mathrm{\pi }}}{2}}(x,y,z)}}{{{\mathrm{I}}_{\frac{{\mathrm{\pi }}}{2} \vdots {\mathrm{max}}}}} \cdot \frac{{{\mathrm{I}}_{\mathrm{\pi }}(x,y,z)}}{{{\mathrm{I}}_{{\mathrm{\pi }} \vdots {\mathrm{max}}}}}]_{{\mathrm{max}}}}}$$

Through this cross-correlation approach, the intensity signal emanating from the LDs was considerably enhanced with respect to the cell cytosol and cell-wall. This level of enhancement enabled the automated localization of LDs by intensity-based thresholding through the maximum-entropy algorithm (ImageJ). Under optimal, low-noise, imaging conditions, this approach yielded a success rate greater than 86% in comparison to the ground-truth, which was established by manually detecting the LD location and size. To correct for the remaining 14%, manual curation was enabled via a Matlab graphical user interface, following similar procedures in cell tracking and lineage reconstruction procedures^67^. On average, the throughput rate of this image analysis step was 2–4 h per experiment.

The last step in the image processing procedure was to convert the acquired phase-delay values of the lipid droplets (ΔΦ_TAG_) and the cytosol (ΔΦ_cytosol_) of individual cells to their corresponding dry-mass (DM) values. For the cytosolic DM we employed the following expression:^24–29^

2 $${\mathrm{DM}}_{{\mathrm{cytosol}}} = \frac{{\mathrm{\lambda }}}{{2 \cdot {\mathrm{\pi }} \cdot \frac{{{\mathrm{dn}}}}{{{\mathrm{dc}}}}}} \cdot \mathop {\int }\nolimits \Delta \phi _{{\mathrm{cytosol}}} \cdot {\mathrm{d}}A$$

Where dn/dc is the protein-specific refractive index increment with a value of 1.85 × 10^−4^ m^3 ^kg^−1^. ΔΦ_cytosol_ is the experimentally measured phase-delay from the cell cytosolic area, *λ* is the wavelength of illumination (centered at 500 nm), with the integration taking place across the entire cytosolic area *A*.

Unlike the cytosolic DM that can be approximated as a protein solution, the specific refractive index increment cannot be directly applied to the LDs^26^. This is because glycerolipids are hydrophobic and, as such, cannot be approximated as a solution in the aqueous cytosolic environment. In contrast, glycerolipids self-assemble as spherical organelles that specifically for *Yarrowia lipolytica* are relatively homogeneous with approximately 90–95% triacylglycerides (TAGs)^35^. This representation was supported by our NanoSIMS analysis, which revealed that LDs are indeed tightly packed with carbon, as illustrated in Fig. 3. Therefore, we employed the Clausius-Mossotti equation to determine the number-density of TAG molecules *N* through the experimentally determined refractive index of the LDs via the following expression:^68^

3 $$N = \frac{3}{{4\cdot {\mathrm{\pi }}\cdot {\mathrm{\alpha }}}}\left( {\frac{{n_0^2 - 1}}{{n_0^2 + 2}}} \right)$$

Where *α* is the TAG molecular polarizability, and *n*_o_ is the refractive index of the LDs. Specific to *Y. lipolytica*, we considered a mixture of 43% triolein, 24% stearin, 20% tripalmitin, 9% trilinolein, and 4% tripalmitolein in accordance to previous reports^35^, yielding a mixture polarizability parameter value of *α*_mix_ = 1.04816 × 10^−22^ cm^−3^. The partial polarizabilities of the mixture components were collected from existing databases^69^. Subsequently, the number density was converted to the LD mass-density *ρ* through the following expression:

4 $${\mathrm{\rho }} = \frac{{N \cdot M}}{{N_{\mathrm{A}}}}$$

Where *N* is the experimentally derived TAG number density, *M* is the TAG molecular weight, *N*_A_ is the Avogadro’s number, and *ρ* is the LD mass density. The molecular weight of the TAG mixture was taken as *m*_mix_ = 1.4375 × 10^−21^ gr, also determined from existing databases^69^.

The selected mixture polarizability (*α*_mix_) and molecular weight (*m*_mix_) parameter values were compared with values corresponding to different LD compositions reported in the literature for a wide range of substrates and *Y. lipolytica* strains^31,35,40^. By comparing more than 20 different strains and growth conditions (including substrate and growth time), we found that the selected *α*_mix_ parameter value differs on average by 0.6% (maximum difference: 1.8%) with the computed *α*_mix_ parameters, while sensitivity analysis yielded the dependence of the selected *α*_mix_ parameter value on different LD compositions as negligible (Supplementary Table 1). To a similar end, the selected *m*_mix_ parameter value was found to change on average by 0.6% (maximum change: 1.6%) with the computed *m*_mix_ parameters (Supplementary Table 2). Overall, this analysis indicates that the selected *α*_mix_ and *m*_mix_ parameter values are not expected to vary significantly under typical growth and substrate conditions for *Y. lipolytica*.

Finally, to attain the LD dry-mass, we multiplied the LD mass density with the LD volume. The latter was determined from the 3D imaging data using the 2D maximum projection method. By fitting a circle to the pixels of the 2D matrix corresponding to the LDs, the LD volume was reconstructed. According to our recent analysis, the 2D maximum projection analysis coupled to fitting yields LD volumes that are equal to those determined directly with spin-disk confocal microscopy^9^ (Spearman correlation coefficient of 0.982, *p* < .001). It is worth adding that the above-described approach faithfully captures the LD corresponding pixels, thus precisely informing about the LD refractive index and size. This is because LDs exhibit higher optical-phase delay than the surrounding cell cytosol. In this way, maximum projection eliminated contributions to the LD signal from the cytosolic biomass (non-TAG) above and below the LDs.

### Strains and growth conditions

Two genetically similar *Y. lipolytica* strains were used in this study, both previously characterized in the context of their lipid metabolism^35^. The Po1g strain is auxotrophic for Leucine (Leu^−^), and it was obtained from Yeastern Biotech Company (Taipei, Taiwan). The MTYL038 strain was constructed to constitutively express the LEU2 gene, which encodes for beta-isopropylmalate dehydrogenase–IMDH, catalyzing the third step in the leucine biosynthesis pathway. LEU2 was expressed under the intronless translation elongation factor-1α promoter. The strains used in this study were Po1g (MATa, leu2-270, ura3-302::URA3, xpr2-332, axp-2) and MTYL038 (MATa, leu2-270, ura3-302::URA3, xpr2-332, axp-2 TEF-LacZ-LEU2).

For the rich YPD medium, we mixed 20 g/L Bacto Peptone (BD), 10 g/L yeast extract (Alfa Aesar), and 20 g/L glucose (Fisher)^35^. Defined YSM medium was made with 1.7 g/L yeast nitrogen base without amino acids and without ammonium sulfate (BD Difco), 0.69 g/L complete supplement mixture (CSM) without Leucine (Sunrise Science Products), and 1.1 g/L ammonium sulfate (Fisher). To synthesize YSM medium with C/N: 15 and 150, 7.5 g/L and 75 g/L glucose (Fisher) was added, respectively. For the Po1g strain, 0.1 g/L Leucine was added in the YSM medium^35^.

All precultures were stored in YPD agarose (Invitrogen) plates at 4 °C and passed twice in YPD medium (5 ml round bottom polystyrene tubes). The second passage was performed at 50 × dilution, followed by a 24 h long growth in YPD, centrifugation at 490 × *g*, and washing in YSM three times, prior to transferring to 125 ml glass shaker flasks (Corning) containing 20 ml of YSM medium. The flasks were covered with polypropylene closures (Corning). The closures were inserted in the open position to enable gas exchange and covered with aluminum foil. The transfer from YPD to YSM was performed at dilutions yielding starting ODs close to 0.01. All experiments were performed using three independent cultures. For this, each of the triplicate cultures was passed from a plate to an independent medium containing tube (for YPD) or flask (for YSM). Each replicate was passed with a 2 h difference from the previous one to facilitate quantitative mass imaging at identical timepoints. All growth experiments were performed in a shaking incubator at a temperature of 29 °C. Growth curves were determined via optical density measurements with a spectrophotometer at a wavelength of 600 nm (VWR, V-1200). To measure the OD curves, cell culture aliquots were introduced in 1 ml polystyrene cuvettes. The resulting growth curves for all growth conditions, strains, and replicates are displayed in Supplementary Figs. 6 and 7.

### Sample preparation

To perform the quantitative mass imaging, we introduced 2 µL from growing cultures between two coverslips and pressed gently to minimize the distance between the two coverslips prior to transferring to the microscope for imaging. Through this approach, cell motion and drift along the XY plane and the optical imaging axis were minimized during image acquisition. For fluorescent imaging, the bodipy dye (BODIPY® 493/503 (4,4-Difluoro-1,3,5,7,8-Pentamethyl-4-Bora-3a,4a-Diaza-s-Indacene–Molecular Probes) solution in DMSO (Molecular Probes) was added in the culture at a concentration of approximately 250 ng/ml, followed by a 25–30 min long incubation under the same temperature and shaking conditions as those during batch growth.

### Nanoscale secondary mass spectrometry

Nanoscale secondary mass spectrometry (NanoSIMS) images were collected at the Environmental Molecular Sciences Laboratory (EMSL) at the Pacific Northwest National Laboratory (PNNL) using a Cameca NanoSIMS 50L. All quantitative mass images were manually analyzed with the OpenMIMS ImageJ plug-in. Stable isotopes were obtained from Cambridge Isotopes, Inc (D-glucose U-^13^C_6_). Samples were coated with 10 nm of high purity gold to improve conductivity (208 h, Cressington Scientific Instruments). Analysis areas were pre-sputtered with 10^16^ ions × cm^−2^. Ion images of ^12^C^−^, ^13^C^−^, ^12^C_2_^−^, ^12^C^13^C^−^, ^12^C^14^N^−^, ^12^C^15^N^−^, and ^31^P^−^ were acquired using a 16 keV, 2 pA Cs^+^ primary beam with a diameter of ∼100 nm. Images were acquired at 512 × 512 pixel resolution with a dwell time of 6.75 ms/pixel. The carbon-13 content was standardized daily using images of an in-house yeast standard (δ^13^C = −11‰ relative to VPDB) acquired in a similar fashion to those of the sample.

For sample preparation, cells were transferred from a plate stored at 4 °C and grown twice in YPD for 24 h (2nd passage at a 50× dilution) in 5 ml round bottom polystyrene tubes appropriately cupped to enable passive gas exchange. Following washing (2×) in isotopic medium by spinning at 490 × *g* for 5 min, cells were then passed to 40 ml of isotopic YSM medium at 10× dilution. To minimize signal saturation, C/N ratios of 15 and 40 were employed, following the medium preparation procedure described in previous sections with isotopically labeled glucose being the sole carbon source. Specific to the C/N: 40, isotopic glucose was introduced at a 20 g/L concentration.

Cells were sampled at various periods ranging from 6 h to 100 h, followed by overnight fixation in 2.5% glutaraldehyde at 4 °C. Subsequently, the samples were washed three times in non-isotopic medium and stained with 1% osmium tetroxide (Ted Pella Inc.) for 1 h. Following three washes in non-isotopic medium, cells were gradually dehydrated in ethanol series at 25%, 33%, 50%, 75% for 15 min each, and 5× in 100% ethanol on a gentle shaker for 30 min Infiltration in the LR White acrylic resin (Ted Pella Inc.) was first performed in a 50:50 mixture with ethanol for 30 min, followed by three washes in 100% resin for 1 h each on a slow rotator. Curing was performed in Eppendorf tubes at 60 °C for 24 h. For imaging, the polymerized blocks were sectioned to 300 nm thin sections with a Leica Ultracut UCT ultra-microtome. Sections were mounted on formvar-coated 100 mesh Cu TEM grids and imaged on Tecnai T-12 TEM (FEI) with LaB6 filament operating at 120 kV. Images were collected digitally using an Ultrascan 1000 CCD (Gatan). The same grids were employed in NanoSIMS imaging.

To confirm that the cytosolic locations exhibiting enhanced ^13^C content correspond to LDs, we undertook a correlative imaging approach using NanoSIMS and TEM for a few select samples. To this end, LDs were first identified in TEM via their osmium mediated increased contrast, and the same cells were imaged in NanoSIMS to determine their elemental composition (Supplementary Fig. 1). In this way, we confirmed that LDs exhibit low content of naturally abundant N, but significantly enhanced ^13^C content, as shown in Fig. 3.

### Statistics

The Kolmogorov-Smirnov statistics were computed in Matlab (Matlab R2018a, Mathworks) using the ktest2 function. 1D and 2D probability distributions were obtained in OriginPro (Origin Pro 2017 64-bit, OriginLab) and through the Kernel Bandwidth optimization approach^70^, respectively. Repeated measures ANOVA tests and non-linear concatenated fits were performed in OriginPro. ANOVA and *t*-tests were performed in Matlab using the anova1 and ttest functions. For the ANOVA, repeated-measures ANOVA, and *t*-tests, the median response from each biological replicate was used. Subpopulation analysis was performed in Matlab using the shifthd function^50^. Sample size selection was guided by previous NanoSIMS^71^, fluorescence image cytometry^9^, and quantitative-phase imaging^24–29^ investigations.

### Reporting summary

Further information on experimental design is available in the Nature Research Reporting Summary linked to this article.

## Supplementary information

Supplementary Information file

Reporting Summary

Source Data

## Data Availability

Data supporting the findings of this work are available within the paper and its Supplementary Information files. A reporting summary for this Article is available as a Supplementary Information file. The datasets generated and analyzed during the current study are available from the corresponding author on reasonable request. The source data underlying Figs. 6b, 7a, 7b, 8a, and 8b, as well as Supplementary Figs. 3, 9, and 10 are provided as a Source Data file.
