# Supplementary material for: Eliciting the impacts of cellular noise on metabolic trade-offs by quantitative mass imaging
Source: Nat Commun. 2019 Feb 19;10:848. doi: 10.1038/s41467-019-08717-w (PMC6381102; doi:10.1038/s41467-019-08717-w)
Supplement: Supplementary file 1 — Supplementary Information file [file 41467_2019_8717_MOESM1_ESM.pdf]

Eliciting the impacts of cellular noise on metabolic trade-offs by quantitative mass imaging

Vasdekis *et al.*

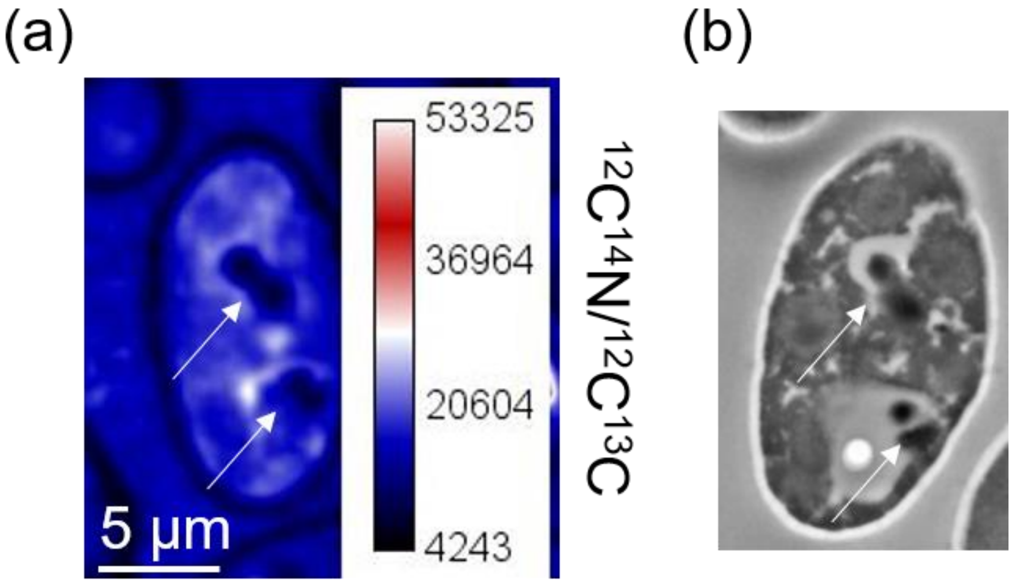

Supplementary Figure 1. Correlative NanoSIMS and TEM imaging for *Y. lipolytica*. **(a)** The $^{12}C^{14}N/^{12}C^{13}C$ ratio image of a representative MTYL038 cell, where lipid droplet (LDs) assemblies, indicated by the white arrows, exhibit low $^{12}C^{14}N/^{12}C^{13}C$ ratios. **(b)** Transmission electron microscopy image of the same cell shown in **(a)**, confirming the position of the LD assemblies by osmium staining (also indicated by white the arrows).

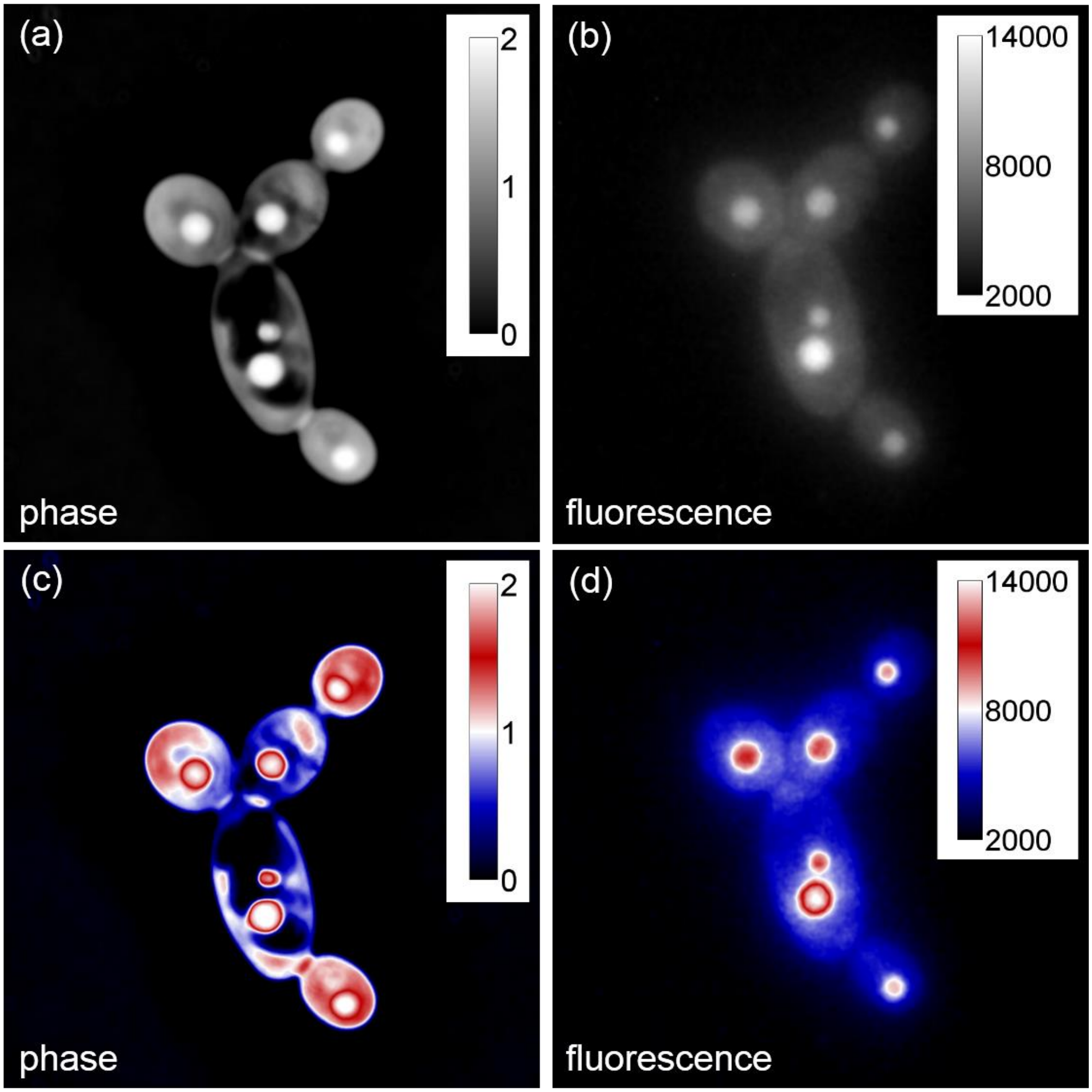

Supplementary Figure 2. Correlative quantitative-phase and fluorescent images for *Y. lipolytica*. (a, c) Phase- and **(b, d)** fluorescent images of the *Y. lipolytica* MTYL038 strain shown also in **Figure 2a**. Comparing the two imaging modalities indicates the agreement between phase and fluorescent LD localization. An independent analysis of 600 single-cell observations yielded a 0.98 Spearman correlation coefficient ($p<.001$) between LDs enumerated in phase and fluorescence imaging. We attribute the imperfect correlation to minor discrepancies emanating from the fluorescent staining procedure.

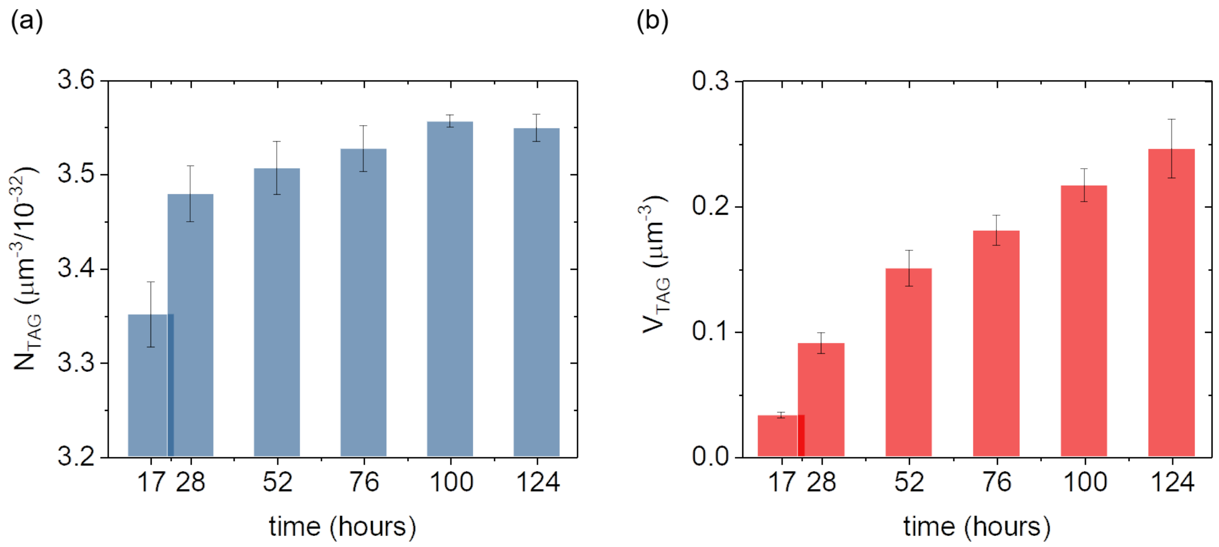

Supplementary Figure 3. Lipid droplet TAG number density and volume dynamics. (a) The evolution of the TAG molecule number density (N_{TAG}) per lipid droplet (LD) for the MTYL038 *Y. lipolytica* strain at 17 hr, 28 hr, 52 hr, 76 hr, 100 hr, and 124 hr of batch growth at a C/N: 150. **(b)** The volume of individual LDs (V_{TAG}) for the same conditions as in **(a)**. Bars and error-bars indicate the mean and standard-error between three biological replicates respectively, based on the following number of observations per timepoint: n_{17} = 2720, n_{28} = 4027, n_{52} = 4561, n_{76} = 4044, n_{100} = 3986, n_{124} = 3767. Source data are provided as a Source Data file.

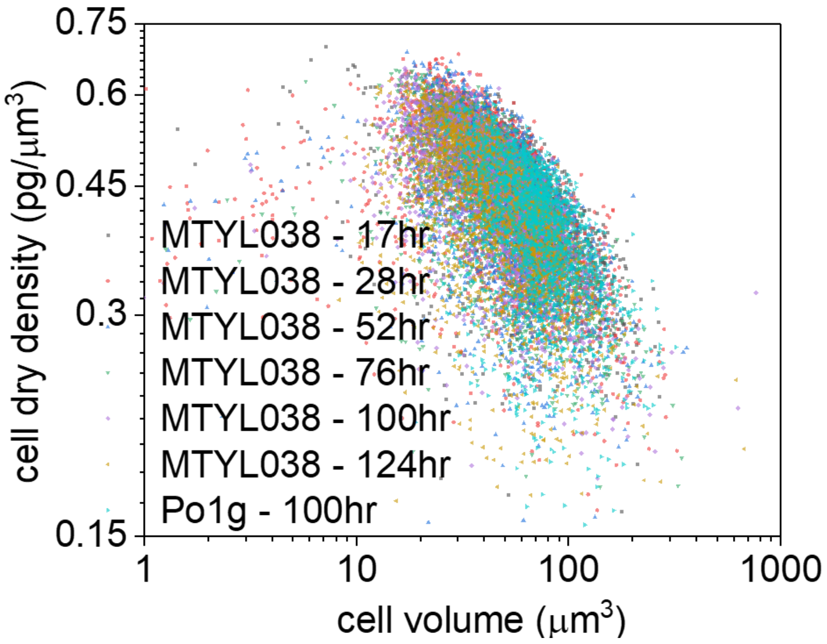

Supplementary Figure 4. Scatter plot of the cell dry-density as a function of the cell volume. Different colors correspond to the MTYL038 *Y. lipolytica* strain grown at C/N: 150 and sampled at 17 hr, 28hr, 52 hr, 76 hr, 100 hr, and 124 hr. Po1g grown at C/N: 150 for 100 hr is also plotted. All data represent three independent cultures per experimental condition, as further detailed in the **Methods** section, with the following total number of observations per condition: n_{M17} = 1351, n_{M28} = 1916, n_{M52} = 1870, n_{M76} = 1991, n_{M100} = 2190, n_{M124} = 2342, n_{P100} = 2110. No evidence was found that the cell volume-density correlation depends on growth and strain conditions (one-way ANOVA with $F(6,14) = 1.16$ and $p=0.38$ between the correlation coefficients of each replicate per experimental condition).

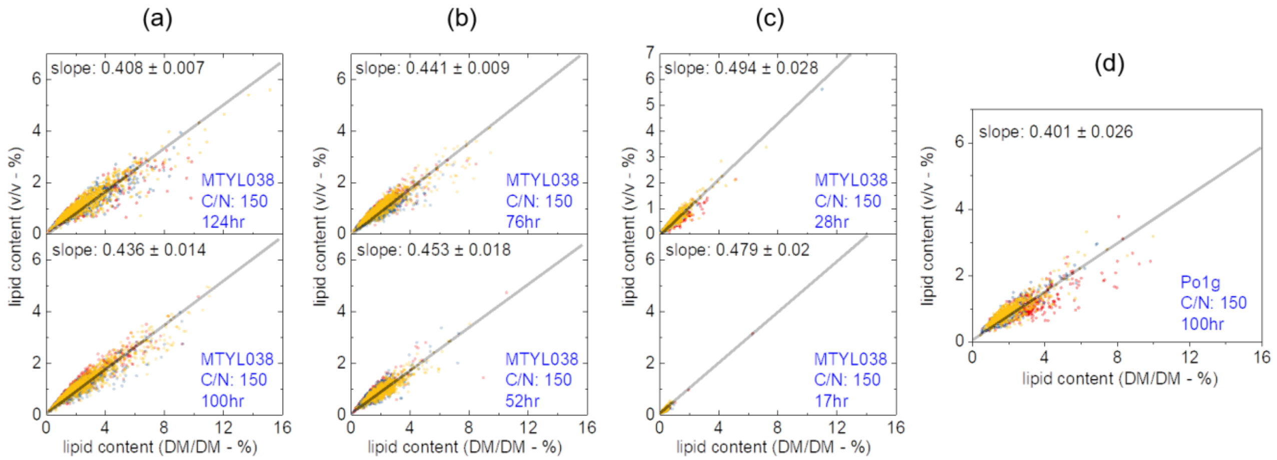

Supplementary Figure 5. Comparison of lipid content in volume and dry-mass. Scatter plots comparing the lipid content in volume (v/v – %) and dry-mass (DM/DM – %) ratios for different strains and growth conditions, specifically. (**a**-**c**) display MTYL038 grown at C/N: 150, sampled at different time points, and (**d**) displays Po1g grown at C/N: 150. All experiments were performed in three biological replicates (number of observations per replicate described in the caption of **Figure 5**), with each replicate represented in a different color. For all tested conditions, slope values are displayed in the legend, represented as the average and standard-error between the three replicates. All slope values were found to be lower than 1 (one-sided t-test, $p<0.03$), evidencing that the lipid content in volume ratios generally underestimates the DM ratios. Further, some growth and strain conditions exhibited higher slopes than others, such as the higher slope of $MTYL038_{28}$ than $Po1g_{100}$ and $MTYL038_{124}$ (one-sided t-test $p<0.03$).

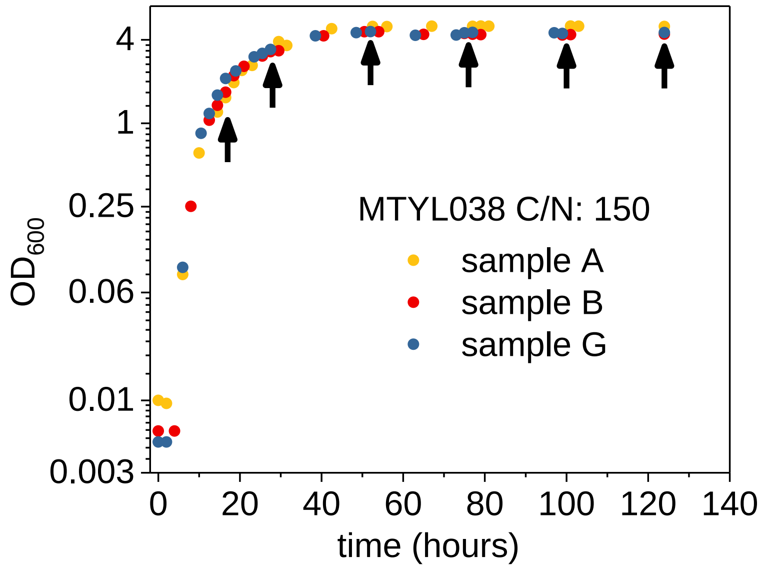

Supplementary Figure 6. Growth curves of *Y. lipolytica* MTYL038. MTYL038 was grown in YSM at C/N: 150 (see **Methods** section for medium details). Black arrows indicate the time-point when cells were sampled for imaging, namely: mid-exponential phase (17 hr), early stationary phase (28 hr), stationary phase (52 hr to 100 hr), and late stationary phase (124 hr). Each replicate (i.e. sample A, B, and G) is color-coded.

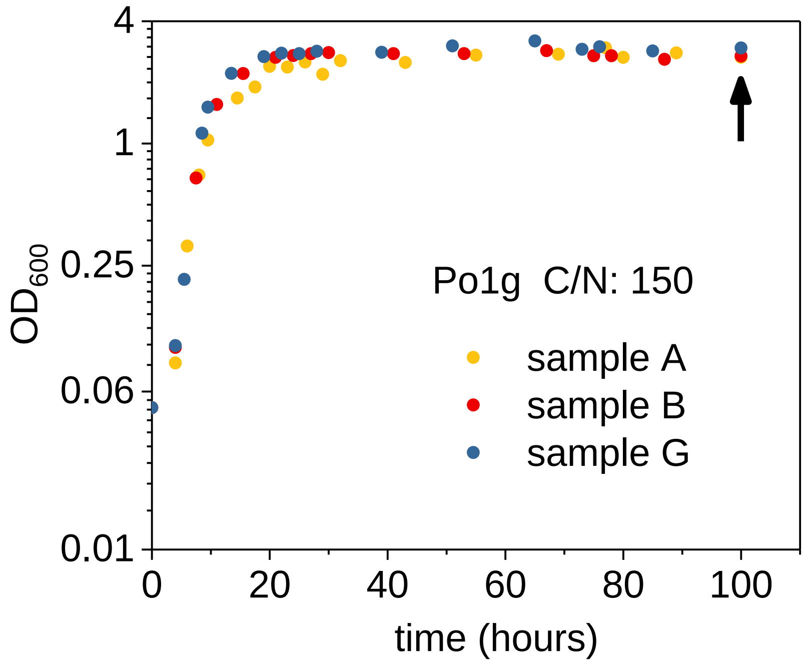

Supplementary Figure 7. Growth curves of the Po1g *Y. lipolytica* strain. Po1g was grown in YSM at C/N: 150 +Leu. Black arrow indicates the 100 hr time-point when cells were sampled for imaging. Each replicate (i.e. sample A, B, and G) is color-coded.

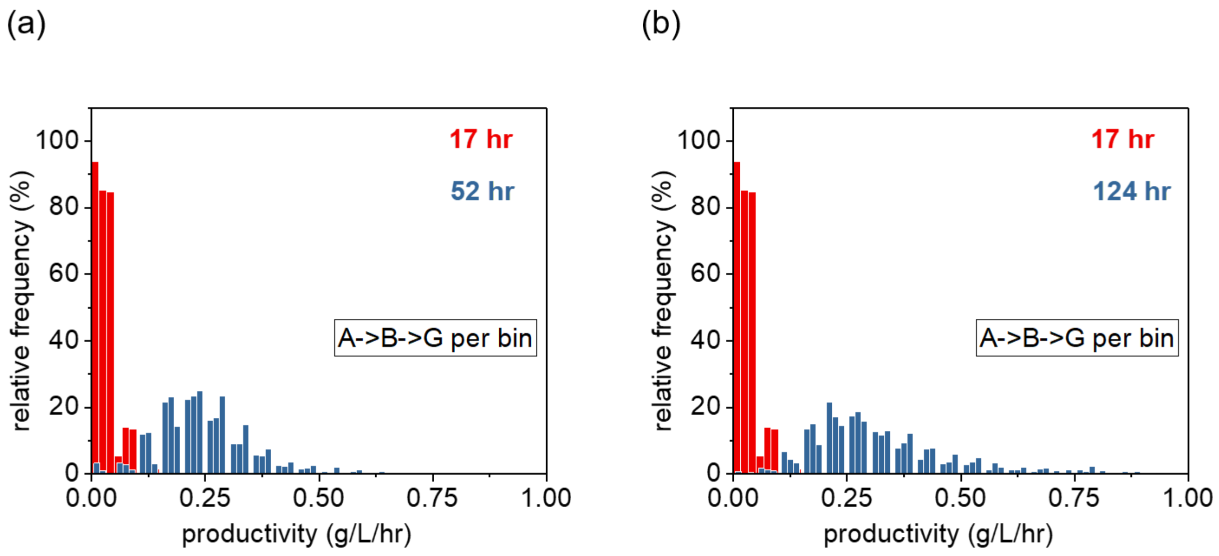

Supplementary Figure 8. Single-cell productivity distributions for *Y. lipolytica* strain MTYL038. MTYL038 was grown in YSM at C/N: 150 at the 17 hr (red) and 52 hr (blue) timepoints (**a**), as well as 17hr (red) and 124 hr (blue) timepoints (**b**). Replicates A, B, and G are sequentially plotted from left to right in each bin.

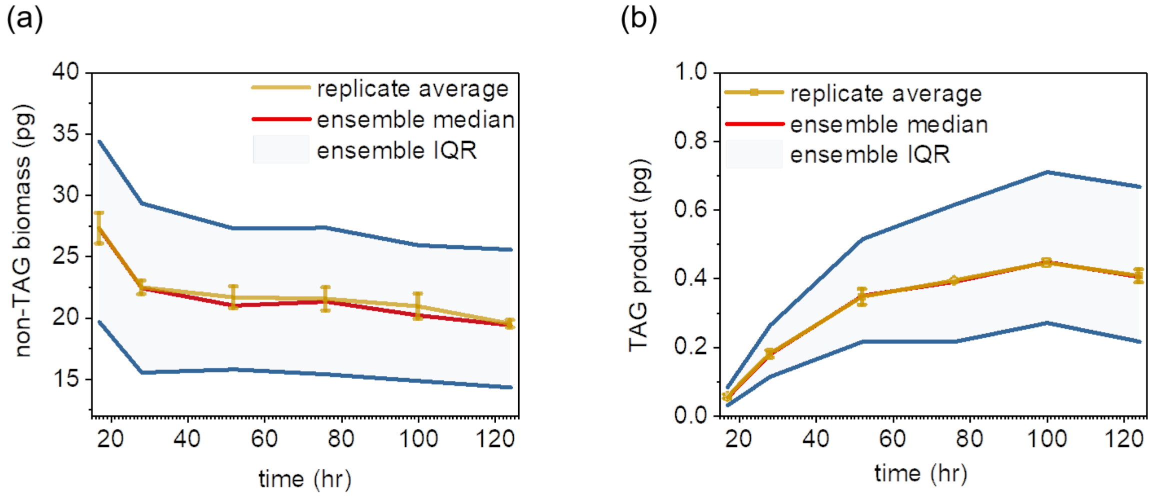

Supplementary Figure 9. Time dependence of the non-TAG biomass and TAG product dry-mass for the *Y. lipolytica* MTYL038 strain. MTYL038 was grown in YSM at C/N: 150. Non-TAG dry-mass is displayed in **(a)**, while TAG-product dry-mass is displayed in **(b)**. The red line and shaded area represent the median and inter-quantile range (IQR) of the ensemble distributions of the three biological replicates. The yellow line represents the average and standard-error (as an error bar) between the median responses of each biological replicate. A statistically significant effect of time was noted for both data sets in (**a**) and (**b**) using repeated measures ANOVA (Greenhouse Geiser adjusted p_{GG}=0.02 with adjustment ε_{GG}=0.290 for non-TAG, as well as p_{GG}<0.001 with ε_{GG}=0.298 for TAG). Figure (**a**) indicates that biomass (non-TAG) decreases throughout the experiment, including a statistically significant autophagy-driven decrease (one-sided t-test p=0.03) after cell-doubling was completed at 52 hr (**Supplementary Figure 6**). Similarly, Figure (**b**) indicates that while the TAG content increases during early stationary phase, it exhibits a statistically significant decrease (one-sided t-test p=0.05)

in late stationary phase, i.e. during the 100 hr – 124 hr period (**Supplementary Figure 6**). Source data are provided as a Source Data file.

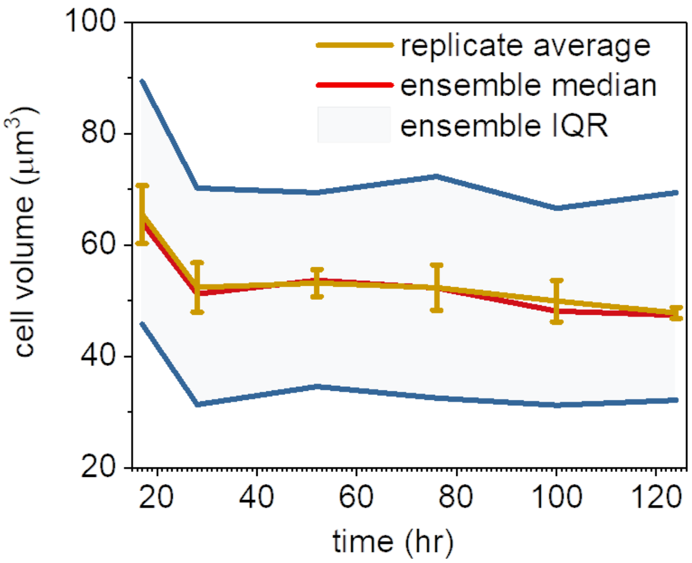

Supplementary Figure 10. Time dependence of the cell volume for the *Y. lipolytica* MTYL038 strain. MTYL038 was grown in YSM at C/N: 150. The red line and shaded area represent the median and inter-quantile range (IQR) of the ensemble distributions of the three biological replicates. The yellow line represents the average and standard-error (as an error bar) between the median responses of each biological replicate. No evidence was found to support that cell volume changes with time (repeated-measure ANOVA p_{GG}=0.15 with ε_{GG}=0.280), contrary to the dry-mass dynamics (p_{GG}=0.02, ε_{GG}=0.290) reported in **Supplementary Figure 9a**. Source data are provided as a Source Data file.

Supplementary Table 1. Lipid droplet mixture polarizability parameter dependence on growth conditions for *Y. lipolytica*.

Reference	α_{mix} (cm^{-3})	% change	sensitivity[1]	medium	time
This work	1.04877E-22	-	-	YSM (glucose)	varying
[2]	1.04665E-22	0.202	negligible	N-lim (glucose)	120 h
[2]	1.03512E-22	1.319	negligible	C-lim (glucose)	120 h
[3]	1.03323E-22	1.504	negligible	YSM (glucose)	120 h
[3]	1.03911E-22	0.929	negligible	YSM (glucose)	120 h
[4]	1.04343E-22	0.511	negligible	27.8 % glycerol	28 h
[4]	1.04106E-22	0.740	negligible	27.8 % glycerol	48 h
[4]	1.04038E-22	0.806	negligible	27.8 % glycerol	130 h
[5]	1.04186E-22	0.663	negligible	YPO (olive oil)	28 h
[5]	1.04822E-22	0.052	negligible	YPO (olive oil)	15 h
[5]	1.05366E-22	0.464	negligible	YPO (olive oil)	10 h
[5]	1.05187E-22	0.295	negligible	YPDO (glucose+oliv.oil)	66 h
[5]	1.06554E-22	1.574	negligible	YPDO (glucose+oliv.oil)	5 h
[5]	1.06838E-22	1.836	negligible	YPDO (glucose+oliv.oil)	10 h
[5]	1.05541E-22	0.629	negligible	YPDO (glucose+oliv.oil)	15 h
[5]	1.06457E-22	1.485	negligible	YPDO (glucose+oliv.oil)	28 h
[5]	1.04963E-22	0.083	negligible	YPDO (glucose+oliv.oil)	46 h
[6]	1.04026E-22	0.818	negligible	fed-batch (FB, glycerol)	-
[6]	1.04137E-22	0.711	negligible	stepwise cont. FB (SCFB, glycerol)	-
[6]	1.04132E-22	0.715	negligible	chemostat (glycerol)	-
[7]	1.05769E-22	0.844	negligible	glycerol	144 h
[8]	1.05203E-22	0.310	negligible	nitrogen-sufficient (glucose)	20 h
[8]	1.05184E-22	0.292	negligible	nitrogen-limiting (glucose)	20 h
[9]	1.04427E-22	0.430	negligible	YSM (glucose)	-
[10]	1.04673E-22	0.194	negligible	YSM (glucose)	-
[10]	1.04823E-22	0.051	negligible	YSM (glucose)	-

The TAG mixture polarizability parameter (α_{mix}) of LDs of different *Y. lipolytica* strains (not shown) grown under different conditions. The % change and sensitivity characterization in comparison to the α_{mix} parameter value used in this work are displayed in the 3rd and 4th columns, respectively.

Supplementary Table 2. Lipid droplet mixture molecular weight dependence on growth conditions for *Y. lipolytica*.

Reference	m_{mix} (g)	% change	medium	time
This Work	1.4375E-21	-	YSM (glucose)	varying
[2]	1.4305E-21	0.490	N-lim (glucose)	120 h
[2]	1.41711E-21	1.439	C-lim (glucose)	120 h
[3]	1.41839E-21	1.347	YSM (glucose)	120 h
[3]	1.42445E-21	0.916	YSM (glucose)	120 h
[4]	1.42932E-21	0.572	27.8 % glycerol	28 h
[4]	1.4261E-21	0.800	27.8 % glycerol	48 h
[4]	1.42472E-21	0.897	27.8 % glycerol	130 h
[5]	1.42927E-21	0.576	YPO (olive oil)	28 h
[5]	1.43703E-21	0.033	YPO (olive oil)	15 h
[5]	1.44291E-21	0.375	YPO (olive oil)	10 h
[5]	1.44098E-21	0.241	YPDO (glucose+oliv.oil)	66 h
[5]	1.45717E-21	1.350	YPDO (glucose+oliv.oil)	5 h
[5]	1.46046E-21	1.572	YPDO (glucose+oliv.oil)	10 h
[5]	1.44524E-21	0.535	YPDO (glucose+oliv.oil)	15 h
[5]	1.45586E-21	1.261	YPDO (glucose+oliv.oil)	28 h
[5]	1.4377E-21	0.014	YPDO (glucose+oliv.oil)	46 h
[6]	1.42613E-21	0.797	fed-batch (FB, glycerol)	-
[6]	1.42731E-21	0.714	stepwise cont. FB (SCFB, glycerol)	-
[6]	1.4268E-21	0.750	chemostat (glycerol)	-
[7]	1.4476E-21	0.697	glycerol	144 h
[8]	1.43774E-21	0.017	nitrogen-sufficient (glucose)	20 h
[8]	1.43771E-21	0.014	nitrogen-limiting (glucose)	20 h
[9]	1.43167E-21	0.407	YSM (glucose)	-
[10]	1.43538E-21	0.148	YSM (glucose)	-
[10]	1.43616E-21	0.093	YSM (glucose)	-

The TAG mixture molecular weight (m_{mix}) of LDs of different *Y. lipolytica* strains (not shown) grown under different conditions. The % change in comparison to the m_{mix} parameter value used in this work is displayed in the 3rd column.

Supplementary Table 3. Lipid content in volume and dry-mass comparison and error quantification.

strain	condition	error (%)
MTYL038	C/N:150 - 17hr	52.1 ± 1.9
MTYL038	C/N:150 - 28hr	50.7 ± 2.8
MTYL038	C/N:150 - 52hr	54.7 ± 1.9
MTYL038	C/N:150 - 76hr	55.9 ± 0.9
MTYL038	C/N:150 - 100hr	56.4 ± 1.4
MTYL038	C/N:150 - 124hr	59.2 ± 0.6
Po1g	C/N:150 + Leu - 100hr	59.8 ± 2.5

Error estimation in determining the lipid content in volume and dry-mass ratios of the lipid product over non-TAG biomass. The error was derived using the slopes of the DM/DM and v/v lipid-content ratios shown in **Supplementary Figure 5**. Errors are reported in the 3rd column as the average and standard-error between the three biological replicates for all tested conditions. Further, some growth and strain conditions exhibited different errors, such as the lower error of $MTYL038_{28}$ than $Po1g_{100}$ and $MTYL038_{124}$ (one-sided t-test $p<0.03$ for both conditions).

Supplementary References

1. Lenhart, T., Eckhardt, K., Fohrer, N. & Frede, H.G. Comparison of two different approaches of sensitivity analysis. *Physics and Chemistry of the Earth, Parts A/B/C* **27**, 645-654 (2002).
2. Kerkhoven, E.J., Pomraning, K.R., Baker, S.E. & Nielsen, J. Regulation of amino-acid metabolism controls flux to lipid accumulation in *Yarrowia lipolytica*. *NPJ Systems Biology and Applications* **2**, 16005 (2016).
3. Tai, M. & Stephanopoulos, G. Engineering the push and pull of lipid biosynthesis in oleaginous yeast *Yarrowia lipolytica* for biofuel production. *Metabolic Engineering* **15**, 1-9 (2013).
4. Makri, A., Fakas, S. & Aggelis, G. Metabolic activities of biotechnological interest in *Yarrowia lipolytica* grown on glycerol in repeated batch cultures. *Bioresource Technology* **101**, 2351-2358 (2010).
5. Najjar, A., Robert, S., Guérin, C., Violet-Asther, M. & Carrière, F. Quantitative study of lipase secretion, extracellular lipolysis, and lipid storage in the yeast *Yarrowia lipolytica* grown in the presence of olive oil: analogies with lipolysis in humans. *Applied Microbiology and Biotechnology* **89**, 1947-1962 (2011).
6. Rakicka, M., Lazar, Z., Dulermo, T., Fickers, P. & Nicaud, J.M. Lipid production by the oleaginous yeast *Yarrowia lipolytica* using industrial by-products under different culture conditions. *Biotechnology for Biofuels* **8**, 104 (2015).
7. Abghari, A. & Chen, S. Engineering *Yarrowia lipolytica* for enhanced production of lipid and citric acid. *Fermentation* **3**, 34 (2017).
8. Zhang, H., Wu, C., Wu, Q., Dai, J. & Song, Y. Metabolic flux analysis of lipid biosynthesis in the yeast *Yarrowia lipolytica* using 13C-labled glucose and gas chromatography-mass spectrometry. *PLOS ONE* **11**, e0159187 (2016).
9. Liu, H.-H. Engineering *Yarrowia lipolytica* for arachidonic acid production through rapid assembly of metabolic pathway. *Biochemical Engineering Journal* **119**, 52-58 (2017).
10. Blazeck, J. et al. Harnessing *Yarrowia lipolytica* lipogenesis to create a platform for lipid and biofuel production. *Nature Communications* **5**, 3131 (2014).
