# Supplementary material for: Eliciting the impacts of cellular noise on metabolic trade-offs by quantitative mass imaging
Source: Nat Commun. 2019 Feb 19;10:848. doi: 10.1038/s41467-019-08717-w (PMC6381102; doi:10.1038/s41467-019-08717-w)
Supplement: Supplementary file 2 — Reporting Summary [file 41467_2019_8717_MOESM2_ESM.pdf]

# nature research

Corresponding author(s): NCOMMS-18-05201D

Last updated by author(s): Jan 20, 2019

# Reporting Summary

Nature Research wishes to improve the reproducibility of the work that we publish. This form provides structure for consistency and transparency in reporting. For further information on Nature Research policies, see Authors & Referees and the Editorial Policy Checklist.

## Statistics

For all statistical analyses, confirm that the following items are present in the figure legend, table legend, main text, or Methods section.

| n/a | Confirmed | |
|---|---|---|
| ☐ | ☒ | The exact sample size ($n$) for each experimental group/condition, given as a discrete number and unit of measurement |
| ☐ | ☒ | A statement on whether measurements were taken from distinct samples or whether the same sample was measured repeatedly |
| ☐ | ☒ | The statistical test(s) used AND whether they are one- or two-sided<br>*Only common tests should be described solely by name; describe more complex techniques in the Methods section.* |
| ☐ | ☒ | A description of all covariates tested |
| ☐ | ☒ | A description of any assumptions or corrections, such as tests of normality and adjustment for multiple comparisons |
| ☐ | ☒ | A full description of the statistical parameters including central tendency (e.g. means) or other basic estimates (e.g. regression coefficient) AND variation (e.g. standard deviation) or associated estimates of uncertainty (e.g. confidence intervals) |
| ☐ | ☒ | For null hypothesis testing, the test statistic (e.g. $F$, $t$, $r$) with confidence intervals, effect sizes, degrees of freedom and $P$ value noted<br>*Give P values as exact values whenever suitable.* |
| ☒ | ☐ | For Bayesian analysis, information on the choice of priors and Markov chain Monte Carlo settings |
| ☒ | ☐ | For hierarchical and complex designs, identification of the appropriate level for tests and full reporting of outcomes |
| ☐ | ☒ | Estimates of effect sizes (e.g. Cohen's $d$, Pearson's $r$), indicating how they were calculated |

*Our web collection on statistics for biologists contains articles on many of the points above.*

## Software and code

Policy information about availability of computer code

| | |
|---|---|
| Data collection | Metamorph Version 7.8.13.0, Cell Vista Pro |
| Data analysis | Origin Pro 2017 64-bit, Matlab R2018a |

For manuscripts utilizing custom algorithms or software that are central to the research but not yet described in published literature, software must be made available to editors/reviewers. We strongly encourage code deposition in a community repository (e.g. GitHub). See the Nature Research guidelines for submitting code & software for further information.

## Data

Policy information about availability of data

All manuscripts must include a data availability statement. This statement should provide the following information, where applicable:

- Accession codes, unique identifiers, or web links for publicly available datasets
- A list of figures that have associated raw data
- A description of any restrictions on data availability

The datasets generated during and/or analysed during the current study are available from the corresponding author on reasonable request.

# Field-specific reporting

Please select the one below that is the best fit for your research. If you are not sure, read the appropriate sections before making your selection.

☒ Life sciences ☐ Behavioural & social sciences ☐ Ecological, evolutionary & environmental sciences

For a reference copy of the document with all sections, see nature.com/documents/nr-reporting-summary-flat.pdf

# Life sciences study design

All studies must disclose on these points even when the disclosure is negative.

| | |
|---|---|
| Sample size | Sample sizes (namely individual cell observations per biological replicate) are typically located in the figure legends. Sample size selection was guided by previous NanoSIMS, fluorescence image cytometry, and quantitative-phase imaging investigations, as referenced in the Methods section. |
| Data exclusions | No data were excluded from the analyses reported in this study. |
| Replication | Each live-cell experiment was performed using 3 independent cultures. All three biological replicates displayed very consistent growth dynamics, determined by their growth curves. All statistics reported in the manuscript are based on these 3 biologically independent replicates. |
| Randomization | Images with single-cell resolution were acquired randomly per experimental condition and biological replicate. |
| Blinding | Single-cell live imaging was performed by automated methods and image analysis was performed by quasi-automated methods. To ensure that the data analysis was unbiased, we first processed all images and then analyzed the results. |

# Reporting for specific materials, systems and methods

We require information from authors about some types of materials, experimental systems and methods used in many studies. Here, indicate whether each material, system or method listed is relevant to your study. If you are not sure if a list item applies to your research, read the appropriate section before selecting a response.

## Materials & experimental systems

| n/a | Involved in the study |
|---|---|
| ☒ | ☐ Antibodies |
| ☐ | ☒ Eukaryotic cell lines |
| ☒ | ☐ Palaeontology |
| ☒ | ☐ Animals and other organisms |
| ☒ | ☐ Human research participants |
| ☒ | ☐ Clinical data |

## Methods

| n/a | Involved in the study |
|---|---|
| ☒ | ☐ ChIP-seq |
| ☒ | ☐ Flow cytometry |
| ☒ | ☐ MRI-based neuroimaging |

## Eukaryotic cell lines

Policy information about cell lines

| | |
|---|---|
| Cell line source(s) | The auxotrophic Po1g (Leu−) strain used in this study was obtained from Yeastern Biotech Company (Taipei, Taiwan). The transformant used in this study (MTYL038) was constructed by the authors and previously reported in: https://doi.org/10.1016/j.ymben.2012.08.007. |
| Authentication | The transformant used in this study (MTYL038) was verified by PCR of prepared genomic DNA when constructed, as reported in https://doi.org/10.1016/j.ymben.2012.08.007. |
| Mycoplasma contamination | Yeast cells used in this study were not tested for mycoplasma contamination. |
| Commonly misidentified lines (See ICLAC register) | Yeast cells used in this study are not listed in ICLAC. |
